# Effect of Huanglongbing on the Volatile Organic Compound Profile of Fruit Juice and Peel Oil in ‘Ray Ruby’ Grapefruit

**DOI:** 10.3390/foods12040713

**Published:** 2023-02-07

**Authors:** Maria Aparecida da Cruz, Anne Plotto, Rhuanito Soranz Ferrarezi, Rui Pereira Leite Junior, Jinhe Bai

**Affiliations:** 1 Agricultural Research Center, University Campus, State University of Londrina, Celso Garcia Cid Rd., Km 380, Londrina 86057-970, PR, Brazil; 2 U.S. Horticultural Research Laboratory, Agricultural Research Service, U.S. Department of Agriculture, 2001 S. Rock Rd., Fort Pierce, FL 34945, USA; 3 Department of Crop Protection, Rural Development Institute of Parana, IAPAR-Emater, Celso Garcia Cid Rd., Km 375, Londrina 86047-902, PR, Brazil; 4 Department of Horticulture, University of Georgia, Athens, GA 30602, USA

**Keywords:** *Citrus paradisi*, gas chromatography–mass spectrometry, hydrodistillation, solid phase microextraction, nootkatone, principal component analysis

## Abstract

Along with orange and mandarin, grapefruit production in Florida has declined sharply due to Huanglongbing (HLB), or citrus greening disease, caused by *Candidatus* Liberibacter asiaticus (*C*Las). HLB affects the volatile profiles of juice and peel oil in oranges, but there is limited information on grapefruit. In this research, ‘Ray Ruby’ grapefruit were harvested in 2020 and 2021 from healthy (HLB−) and HLB-affected (HLB+) trees. Peel oil was extracted by hydrodistillation, and the volatiles were analyzed by direct injection of the oil samples into gas chromatography–mass spectrometry (GC-MS). Volatiles in the juice were analyzed by headspace (HS)-solid-phase microextraction (SPME) coupled with GC-MS. HLB significantly altered the volatile profiles of peel oil and juice in ‘Ray Ruby’ grapefruit. Juice samples of HLB+ fruits had lower decanal, nonanal, and octanal, important citrus juice flavor compounds. HLB+ samples also showed reduced content of nonterpene compounds, other aliphatic and terpene aldehydes, and terpene ketones. Ethanol, acetaldehyde, ethyl acetate, and ethyl butanoate were increased in HLB+ juice samples, indicating an HLB-induced stress response. The most abundant compounds D-limonene and β-caryophyllene, as well as other sesquiterpenes, were increased in HLB+ juice and peel oil samples. On the other hand, the oxidative/dehydrogenated terpenes were increased by HLB in peel oil but decreased in the juice sample. Nootkatone, the key grapefruit volatile was consistently reduced by HLB in both peel oil and juice samples. The impact of HLB on nootkatone deteriorated the quality of both juice and peel oil in grapefruits.

## 1. Introduction

Florida citrus production has plummeted since 2005 when Huanglongbing (HLB), or citrus greening disease, was first reported in the U.S. Total citrus production has been reduced by 80% from 291.8 million boxes in 2003/2004 to 57.8 million boxes last season (2020–2021) [1]. The impact on the grapefruit (*Citrus paradisi*) industry is even more severe, with a decrease of 90% from 40.9 to 4.1 million boxes [1,2]. Citrus canker disease (*Xanthomonas citri* subsp. citri) and associated eradication programs from the late 1990s to the early 2000s followed by catastrophic hurricanes in 2004 and 2005 were also responsible for the decrease in acreage and production [3,4].

HLB is caused by a gram-negative bacterium, ‘*Candidatus* Liberibacter asiaticus’ (*C*Las), which is transmitted by the Asian citrus psyllid (ACP, *Diaphorina citri*) [5]. The disease has spread through the major citrus-producing regions worldwide, causing high economic losses in the citrus industry [4]. HLB seriously affects citrus production because of premature fruit drop, resulting in yield reduction [6,7] and, eventually, tree death [3]. In oranges, fruits of HLB-affected (HLB+) trees usually are reduced in size, remain green, and are asymmetric [8]. The juice of these fruits has lower sugar and soluble solids contents (SSC), higher titratable acidity (TA), a lower SSC/TA ratio, and higher limonoid and flavonoid contents [4]. Among flavor volatiles, ethyl butanoate, valencene, decanal, and other ethyl esters are usually lower, but many monoterpenes are higher in symptomatic fruit compared to healthy and asymptomatic fruit [4]. The juice is less sweet, sourer, bitter, and sometimes has a metallic and off-taste [4,9,10].

Citrus volatile organic compounds (VOCs) include oil-soluble compounds commonly found in the peel and water-soluble compounds in juice [11,12]. Citrus peel oil, a coproduct of the juice industry, is rich in terpene compounds that are used as ingredients in the food and perfume industries [13]. Grapefruit essential oil is well-known for its antioxidant, antiseptic, disinfectant, diuretic, and stimulant properties and is used for different industrial purposes [13,14,15]. Among the reported grapefruit volatiles, nootkatone is the most important and valuable aromatic present in the peel and juice [13,14]. This ketone is very popular in the food and cosmetic industries. It has potential health benefits, such as the prevention of obesity and hyperglycemia, improving physical performance, and possibly having protective activity against learning and memory impairments [16,17].

Previous studies reported changes in the chemical composition of orange peel oil due to HLB infection [18,19,20]. Kiefl et al. [20] showed that HLB+ orange peel oil contained a lower concentration of several long-chain aldehydes, such as octanal and decanal. Xu et al. [18] found that severely HLB+ oranges had a lower abundance in most oxygen-containing volatile components, such as linalool, decanal, citronellol, neral, geranial, carvone, dodecanal, and 2-decenal in the peel oil, compared to less severely HLB+ fruits. However, an aroma sensory panel could not discriminate between the two oils, indicating that HLB severity may not affect flavor [19]. Sun et al. [21] showed that peel oil extracted from HLB+ ‘Valencia’ orange fruits had lower concentrations of typical peel oil components, including valencene, octanal, and decanal, and were abundant in oxidative/dehydrogenated terpenes, such as carvone and D-limonene oxide. However, D-limonene, the dominant component, was not affected by disease status.

Research on the effects of *C*Las bacterium on VOC profiles in citrus is sparse and nonconclusive, and most research is based on sweet oranges [4]. This study aims to evaluate the effect of *C*Las on VOCs in the peel oil and juice of ‘Ray Ruby’ grapefruit.

## 2. Materials and Methods

### 2.1. Site Location, Plant Material, and Cultural Practices

**Figure 1:**
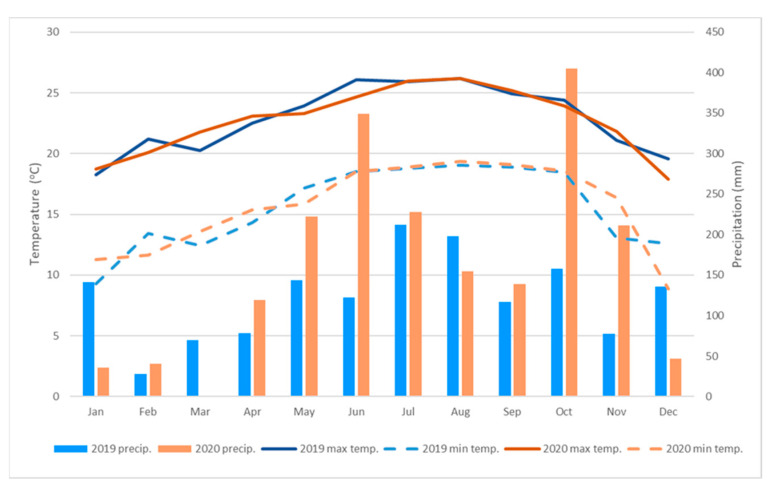
Precipitation, maximum, and minimum temperatures for 2019 and 2020. Fort Pierce, FL, USA. (Source: FLORIDA CLIMATE CENTER, 2021).

‘Ray Ruby’ grapefruit trees grafted on ‘US-897’ [‘Cleopatra’ mandarin (*C. reticulata*) × ‘Flying Dragon’ trifoliate orange (*Poncirus trifoliata*)] rootstock were planted in plastic pots on September 2013 at the UF/IFAS Indian River Research and Education Center in Fort Pierce, FL, USA (lat. 27°26′ N, long. 80°26′ W, 10 m elevation above sea level). Monthly average maximum and minimum temperatures and cumulative precipitation data for the years 2019 and 2020 before the 2020 and 2021 harvests were collected from the U.S. Climate Data (Figure 1) (Station of Ft. Pierce, FL, USA, 10 km from the farm) (https://www.usclimatedata.com/climate/fort-pierce/florida/united-states/usfl0156, accessed on 10 May 2021).

We used 37.85 L plastic pots (#10 Accelerator AP-10; Nursery Supplies, Chambersburg, PA, USA) filled with a medium consisting of (*v*/*v*) 50% clean washed silica sand, 15% Florida peat moss, 7.5% coconut fiber, 20% cypress sawdust, and 7.5% perlite (Harrell’s, Lake Placid, FL, USA). The pots were placed on ceramic tiles to prevent tree roots from growing into the underlying native sandy soil. Each tree in the trial received routine irrigation and fertigation program according to Ferrarezi et al. [22].

Healthy grapefruit trees (HLB−) were cultivated under a protective screen that acted as a barrier against the ACP to prevent any transmission of the disease. HLB+ trees were grown in the open air and naturally exposed to a high ACP population [22]. Each treatment contained 24 trees and was equally divided into three replications.

### 2.2. ‘Candidatus Liberibacter asiaticus’ Detection

Ten fully expanded leaves and leaf petioles with intact stems were collected from eight trees per replication. The detection analysis was conducted by quantitative real-time polymerase chain reaction (qRT-PCR) using the U.S. Department of Agriculture, Animal, and Plant Health Inspection Service approved primers [5,22]. The cycle threshold (Ct) to consider trees infected by HLB was below 32 [5].

### 2.3. Fruit Harvest and Measurements of Fruit Color, Size, Soluble Solids Contents, and Acidity

Fruits were harvested on 8 January 2020 and 7 January 2021. One fruit was harvested per tree, and eight fruits per replicate were collected. Fruits were washed using vegetable detergent, rinsed with deionized water, and air dried at room temperature for 30 min. The color was measured at three points around the fruit equator using a Chromameter (Minolta CR-400, Tokyo, Japan) calibrated to a white plate using the CIELAB system, and a*, b*, a*/b*, and hue values were reported [23]. A higher a*/b* value and/or lower hue value indicates a more orange or red color [6,24,25]. Individual fruit was measured for weight and size. Data reported are an average of eight individual fruits x three replicates.

The flavedo tissue (1–2 mm from the fruit surface) was manually taken using a sharp stainless-steel knife for peel oil extraction. The flavedo-removed fruits were then halved, and juice was gently extracted using a kitchen juicer with an automatic self-reversing reamer (Oster Model 3183, Household Appliance Sales and Service, Niles, IL, USA). Juice samples were centrifuged at 10,000× *g* for 15 min. The supernatant was used for soluble solids content (SSC) and titratable acidity (TA) measurements. SSC was determined by refractive index using a digital refractometer (Atago RX-5000cx, Tokyo, Japan). TA was measured by titration of 10 mL of supernatant with 0.1N NaOH to pH 8.1 using an autotitrator (808 Titrando, Metrohm, Riverview, FL, USA).

### 2.4. Peel Oil Extraction and Gas Chromatography–Mass Spectrometry (GC-MS) Analysis

Peel oil was extracted by hydrodistillation [26]. The peel tissue, 200 g, from eight fruit in each replicate was homogenized in a blender with 500 g of ultrapure water and transferred to a 2000 mL round bottom flask fitted with a heating mantle. The oil sample was collected using a Pyrex Allihn Condenser (Corning, Inc., Corning, NY, USA) and a Clevenger-type receiver trap (Product #LG-11125-102, Wilmad–LabGlass, Vineland, NJ, USA) in a 2 h process until no more oil was obtained. The hydrodistillation system was cooled with an ethylene glycol–water (1:1 *v*/*v*) solution at −3 °C. The distilled oil mixture/emulsion was allowed to settle for 15 min. Then, the oil layer was collected and centrifuged at 8500× *g* at room temperature for 5 min to remove heavy impurities, and to separate it from water. The oil was then dried over anhydrous sodium sulfate and stored at −20 °C until analysis.

The volatile compositions in the oil samples were analyzed using a GC-MS system as previously reported [21]. The GC-MS (6890N GC, and 5975 MS, Agilent Technologies, Santa Clara, CA, USA) equipped with a capillary column (DB-5; 60 m length, 0.25 mm diameter, and 1 µm film thickness, J&W Scientific, Folsom, CA, USA) was used [21]. One microliter of peel oil was injected in a split/splitless injector (250 °C) in the split mode (split ratio 40:1). The oven was held at 40 °C for 0.5 min then increased to 225 °C at 4 °C min^−1^ and held for 13.25 min. The mass spectrometry data were recorded in the scan mode at an ionization energy of 70 eV. The mass range was from 40 to 400 at 2 scans s^−1^. The inlet, ionizing source, and transfer line were kept at 250, 230, and 280 °C, respectively.

### 2.5. Juice Extraction and GC-MS Analysis

VOCs in juice samples were analyzed by headspace solid-phase microextraction (HS-SPME) coupled with GC-MS as described previously [11]. Freshly squeezed juice, 6 mL of each juice sample, was pipetted into a 20 mL vial and crimp-capped with a Teflon/silicone septum. A triplicate of each juice sample was prepared. Sample vials were stored at −20 °C until analysis.

Frozen sample vials were thawed under tap water and loaded into the autosampler (Model MPS2; Gerstel Inc., Linthicum, MD, USA) equipped with a cooled (4 °C) tray holder (Laird Tech, Göteborg, Sweden) controlled by a Peltier thermostat (CTC Analytics AG, Zwingen, Switzerland) for headspace sampling and GC-MS analysis. The analyses were conducted as described by Bai et al. [11].

### 2.6. Identification and Quantification of VOCs

GC-MS data were collected using the ChemStation G1701 AA data system (Hewlett-Packard, Palo Alto, CA, USA). A mixture of C-5 to C-20 n-alkanes were run at the beginning of each day to calculate retention indices (RIs) [11]. The volatile components were identified by matching their spectra with library entries [National Institute of Standards and Technology (NIST)/Environmental Protection Agency (EPA)/National Institutes of Health (NIH) Mass Spectral Library (NIST 14; WebBook, SRD69)] and authentic volatile compound standards, as well as by comparing their RIs with corresponding literature data [27]. For the quantification of the juice samples, peak size (total ion current) was used to compare the relative abundance between samples. To quantify D-limonene, which usually makes up more than 90% of total peel oil, peel oil samples were diluted to 3% with methanol. Standard curves were used to determine the concentration of each compound.

### 2.7. Statistical Analyses

Statistical analysis for fruit color, size, sugar, and acid was performed with statistical software (R version 4.1.0; R Core Team, 2021, http://www.r-project.org, accessed on 6 July 2021) using the ExpDes package. The data were tested for adequacy of normal distribution and homogeneity at 5% probability and evaluated using analysis of variance followed by Tukey’s test (*p* ≤ 0.05). The volatile compounds data were analyzed with JMP software (version 11.2.2; SAS Institute, Cary, NC, USA). Differences were tested using Tukey’s honest significant difference (HSD) (*p* ≤ 0.05). Cluster analysis (CA) and principal component analysis (PCA) were used to characterize the chemical profile of peel oil across HLB+/HLB−.

## 3. Results and Discussion

### 3.1. Huanglongbing Effect on Fruit Size, Color, SSC, and TA

Healthy grapefruit trees (HLB−) cultivated under the protective screen had a healthy canopy, fruits, and leaves, and there was no ACP under the screen [22]. The qPCR results showed that all leaf samples were CLas negative with Ct values > 40. On the other hand, all HLB+ trees grown in the open air were exposed to a high ACP population, and therefore a high CLas pressure, and had a compromised canopy, very few leaves, and greener, smaller, misshapen fruits, all typical symptoms of the HLB disease. The qPCR results showed that Ct values of CLas were 27.3 ± 0.64 in the leaf samples, confirming those trees were all infected by the bacterium [22].

**Figure 2:**
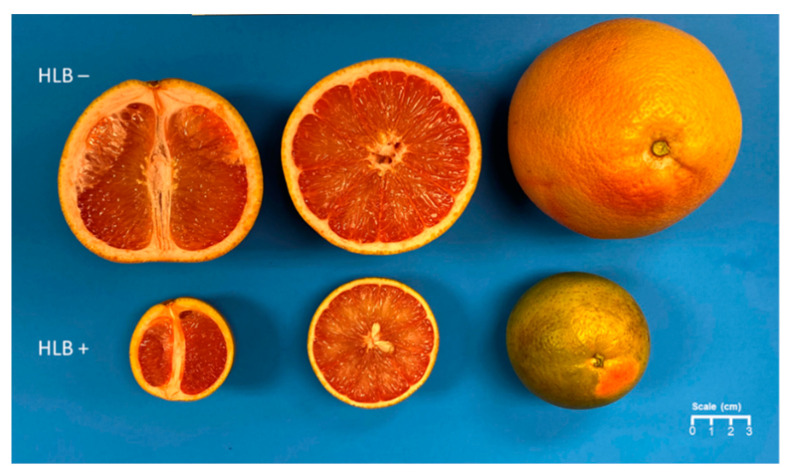
Fruit appearance of ‘Ray Ruby’ grapefruit of healthy (HLB−) and HLB-affected (HLB+) fruits (2021).

HLB+ fruit samples exhibited a lower chromameter a*, a*/b* ratio and higher hue angle than healthy ones, indicating a greener and less orange fruit peel color in both the 2020 and 2021 seasons. Chromameter b* values did not statistically differ between the treatments but were slightly higher for HLB+ samples, confirming a less yellow and more blue peel color in HLB+ fruit samples (Table 1 and Figure 2). In 2021, the HLB+ fruits were less green than the ones of the 2020 season, as revealed by the higher peel a*/b* ratio and lower hue angle (Table 1). Regarding fruit size, HLB+ fruits were smaller in weight, length, and diameter in 2020 and 2021 compared to HLB− ones (Table 1 and Figure 2). The lower fruit size associated with the higher preharvest drop caused by HLB results in low fruit yield [7]. HLB+ grapefruit juice had a statistically reduced SSC and SSC/TA ratio (Table 1). However, there was no significant difference in TA (Table 1). At the point of evaluation, the samples, regardless of HLB+/− and harvest season, met the minimal maturity standards for grapefruit in Florida, USA [28], with the SSC of the juice above 7.5%, the SSC/TA ratio higher than 7.0, and more than 25% of the fruit surface with yellow or red color (Table 1).

Overall, HLB+ fruits were smaller and greener with a lower SSC and SSC/TA ratio (Table 1 and Figure 2). These aspects are characteristics of HLB+ fruit and have been reported by several authors, particularly for sweet oranges, such as ‘Valencia’ and ‘Hamlin’, which were the most studied cultivars for the effect of HLB on fruit quality [4,6,8,10,29]. Usually, as the maturity progresses, the peel color of a healthy orange becomes less green and more orange, and TA in the juice decreases [12]. In citrus peel, chlorophylls are more abundant in dark green fruits. Carotenoids, however, follow the opposite trend [25]. Previous research showed that grapefruits grown inside the tree canopy or bagged accumulated more lycopene and carotenoids and less chlorophyll, which were evidenced by an acceleration of chloroplasts to chromoplast transition in the peel and the relative expression of genes related to chromoplast development was enhanced by light avoidance [30]. HLB+ had a much smaller tree canopy and fewer leaves [4,22], which could be one of the reasons HLB+ fruits were greener. HLB delays fruit development; thus, TA remains at high levels for a longer time. This high TA content is often reported for the juice of HLB+ oranges [4]. However, in this research, HLB did not significantly affect juice TA content in grapefruit juice (Table 1).

### 3.2. Huanglongbing Effects on the Volatile Profile of “Ray Ruby” Grapefruit Peel Oil

A total of 75 VOCs were detected in ‘Ray Ruby’ grapefruit peel oil samples over the two harvest seasons (Table 2 and Figure S1). The compounds included 16 sesquiterpenes, 15 alcohols (2 aliphatics + 13 terpene alcohols), 13 monoterpenes, 8 aliphatic aldehydes, 7 esters, 5 terpene aldehydes, 5 terpene oxides, 4 ketones, and 2 acids (Table 2).

**Figure 3:**
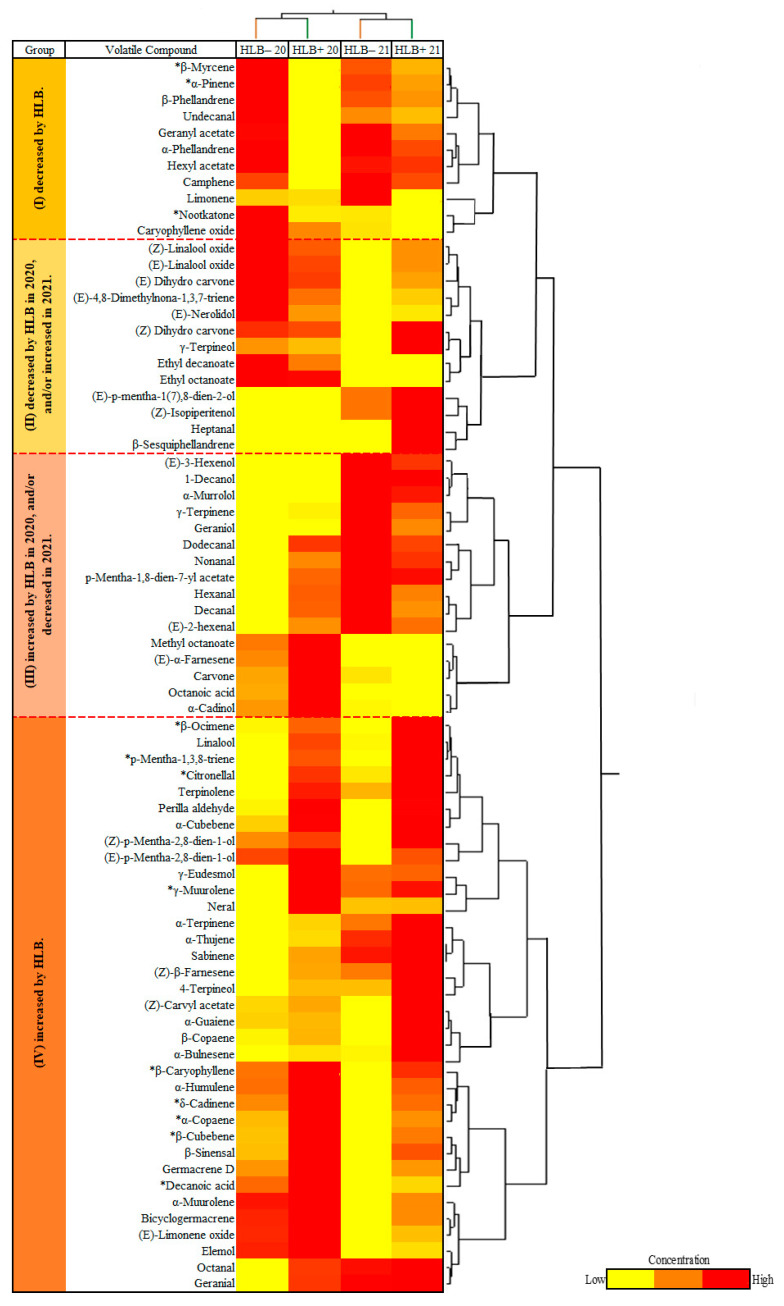
Combined cluster analysis and heat map of grapefruit peel oil samples from healthy (HLB−) and HLB-affected fruits (HLB+) from 2020 (HLB− 20 and HLB+ 20) and 2021 (HLB− 21 and HLB+ 21) harvests (columns) and identified VOCs (rows). Chemical groups increased and/or decreased by Huanglongbing (HLB) determined by the average concentration of volatiles in peel oil extracted from ‘Ray Ruby’ grapefruit in two harvest seasons, 2020 and 2021. * Significant differences at *p* ≤ 0.05 according to Tukey’s HSD test in both years.

**Figure 4:**
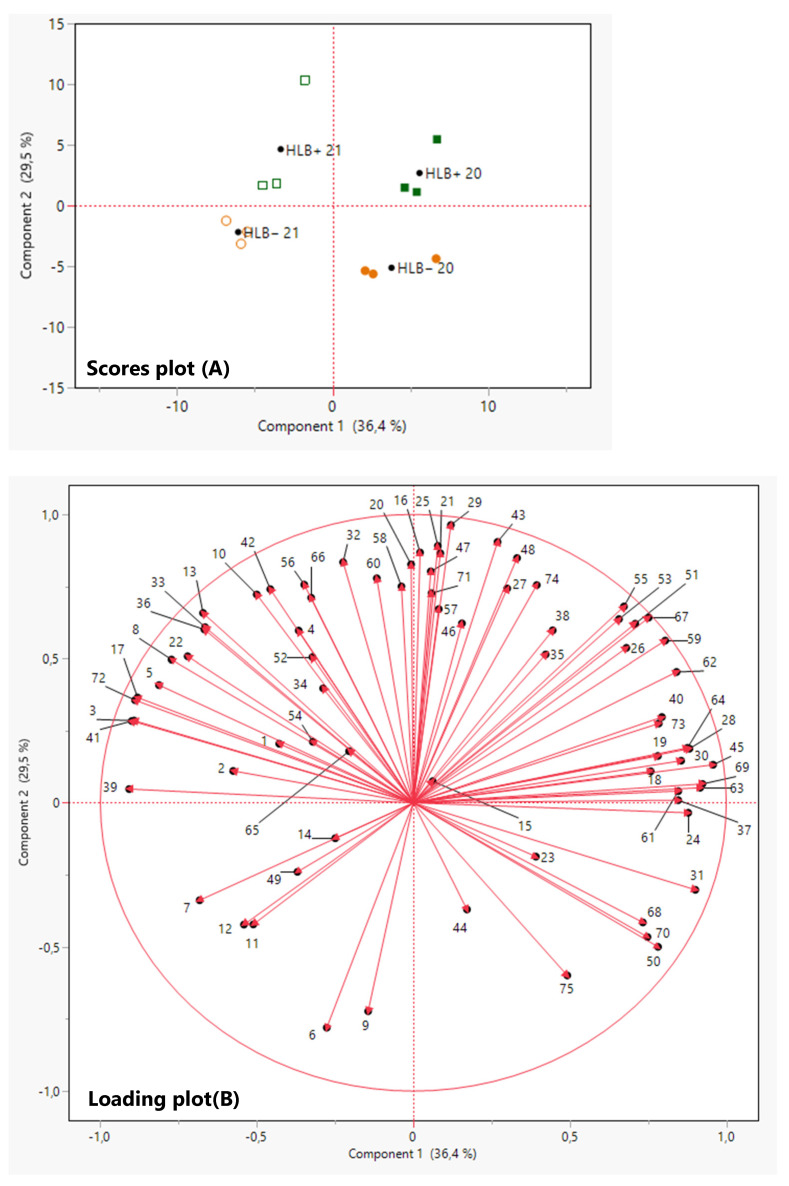
Principal component analysis (PCA) score plot (**A**) and loading plot (**B**) of VOCs in peel oil of ‘Ray Ruby’ grapefruit of healthy (HLB−) and HLB-affected (HLB+) fruits from 2020 (HLB− 20 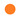 and HLB+ 20 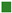) and 2021 (HLB− 21 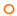 and HLB+ 21 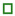) harvests. VOCs corresponding to the numbers are listed in Table 2.

Monoterpene hydrocarbons were the predominant chemical class in all samples regardless of the year and treatment, with the content being 96.68% and 96.81% in HLB+ and 97.11% and 98.20% in HLB− peel oil samples in 2020 and 2021, respectively (Table 2). D-limonene is the main volatile in citrus fruit and is an important contributor to the citrus flavor [31]. CA and PCA analysis showed that D-limonene was associated with HLB− samples (higher concentrations in HLB− samples, Figure 3 and Figure 4). However, there were no significant differences (Table 2). The results agreed with reports on orange peel oils [18,21].

The other abundant compounds in ‘Ray Ruby’ grapefruit peel oil samples were β-myrcene, α-pinene, (E)- β-caryophyllene, and decanal (Table 2). β-Myrcene and α-pinene, major monoterpenes next to D-limonene, were found in significantly higher contents in HLB− samples in 2020 and 2021 (Table 2). This differed from most reports that showed HLB did not significantly affect monoterpene content in orange peel oils [18,21]. These volatiles have been found in high concentrations in citrus peel oils and have a large contribution to the peel oil aroma [32]. β-Myrcene is reported to have a green and mossy odor and is a much stronger odorant than D-limonene [33]. β-Myrcene is a versatile terpene used in different applications, mainly in the perfume and fragrance industries [34]. α-Pinene presents a pine tree resin aroma that can make a positive background contribution to the aroma of the sweet orange [33].

Sesquiterpene hydrocarbons were the second largest class in the peel oil, and the major VOCs in the class were (E)- β-caryophyllene, δ-cadinene, α-copaene, β-cubebene, and γ-muurolene, which were significantly increased by HLB affection in both years (Table 2). Similar observations were reported by Xu et al. [18] and Zhang et al. [35] in orange peel oil; stress from the bacterium preventing nutrient flow to the fruit may cause shifts in the biochemical pathways, creating a higher accumulation of those volatiles in the peel oil [18]. However, Sun et al. [21] reported that HLB decreased sesquiterpenes in orange peel oil.

In the 2020 harvest, HLB significantly increased the total aldehydes content in peel oil. However, there was no difference in the 2021 season (Table 2). The individual analyses of the aldehydes revealed that citronellal, perilla aldehyde, β-sinensal, octanal, geranial, and neral had higher content in HLB+ samples in 2020 or both seasons, while undecanal had lower content (Table 2). Xu et al. [18] also found high citronellal and perilla aldehyde in oils of HLB symptomatic oranges.

However, previous reports showed that HLB significantly reduced most long-chain aliphatic aldehydes, including octanal, nonanal, and decanal, of orange peel oil [4,20,21]. C8-C10 aldehydes are associated with peel oil quality by the pharmaceutical standard [19].

Nootkatone content was consistently higher in HLB− peel oil samples, 7.9- and 2.7-fold higher in 2020 and 2021, respectively (Table 2). Nootkatone is the most important and expensive aromatic compound in grapefruit [15]. In combination with other carbonyl-containing constituents, nootkatone is responsible for the flavor of good-quality grapefruit oil [36]. The concentration of nootkatone is used as the criterion for oil quality [15]. The accumulation of nootkatone in the peel is associated with the processes of maturation and senescence of the fruits [37]. It has been suggested that HLB+ fruits have a slow maturation due to a compromised vascular system, similar to immature fruits [9]. This can explain why the sesquiterpene ketone in HLB+ peel oil was lower than in healthy fruit peel oil. Nootkatone has been isolated from grapefruit peel and juice and can also be obtained from the oxidation of valencene [34,35]. Naturally produced nootkatone has a strong grapefruit odor, while the synthetic form shows a weak woody and spicy flavor, not typical of grapefruit flavor [34]. Grapefruit peel is the main source of nootkatone for the industry, and due to its limited production, it has a very high price [36]. Thus, a lower concentration of this sesquiterpene in the peel oil of HLB+ fruit has a major economic impact.

Previous studies reported that lower concentrations of linalool were found in orange peel oils derived from severely HLB+ fruit [18]. However, in the present study, HLB infection increased the accumulation of this compound in grapefruit peel oil in both seasons (Table 2). This terpene alcohol is an oxygenated product of D-limonene, but unlike limonene, linalool accounts for characteristic citrus and floral odors, an important aroma for citrus [38]. Because of its flavorful and fragrant properties, linalool is used in the food, perfume, and cosmetics industries [39]. Besides that, linalool also exhibits strong antibacterial activity against bacterial strains [26].

A CA divided the compounds into four groups: group I with 11 compounds decreased due to HLB, including nootkatone and two abundant monoterpenes, β-myrcene and α-pinene (Figure 3); group II where 13 compounds were decreased by HLB in 2020 but increased by HLB in 2021; group III with 16 compounds increased by HLB in 2020 but decreased in 2021; and group IV with 35 compounds increased by HLB, including most sesquiterpene hydrocarbons, terpene aldehydes, terpene oxidations, and dehydrogenations (Figure 3). More than 24 compounds were increased by HLB (35 increased–11 decreased, Figure 3). Some of the differences through the years in groups II and III may be due to climatic conditions, as the 2021 season was consistently rainy in the months preceding harvest (Figure 1).

PCA was performed to project the volatile components onto a two-component plot (Figure 4). The PCA discriminated HLB+ from HLB− samples mainly on Component 2, explaining 29.5% of the variation (Figure 4A,B). Volatiles from HLB+ samples were generally on the positive side of PC2, and HLB− samples were on the negative side of PC2 (Figure 4A,B). The loading plot (Figure 4B) in PCA confirmed that more compounds were associated with HLB+ fruits (Figure 4). The PCA discriminated the 2021 season samples from the 2020 season samples mainly on PC1, explaining 36.4% of the variation (Figure 4A,B). The 2020 season had substantially low rainfall (Figure 1), which may have caused more stress, thus producing more oxidative and dehydrogenated terpenes (Table 2 and Figure 4).

### 3.3. Huanglongbing Effects on the Volatile Profile of ‘Ray Ruby’ Grapefruit Juice

A total of 87 volatiles were detected in HLB+ and HLB− ‘Ray Ruby’ grapefruit juice samples over two harvest seasons, and the components were classified into 12 chemical classes (Table 3 and Figure S2). In comparison to peel oil, the VOCs in the juice had a second major compound next to D-limonene, β-caryophyllene, a sesquiterpene hydrocarbon, and contained 12 more VOCs (a 16% increase), which were mostly low molecular weight aliphatic compounds (Table 2 and Table 3).

**Figure 5:**
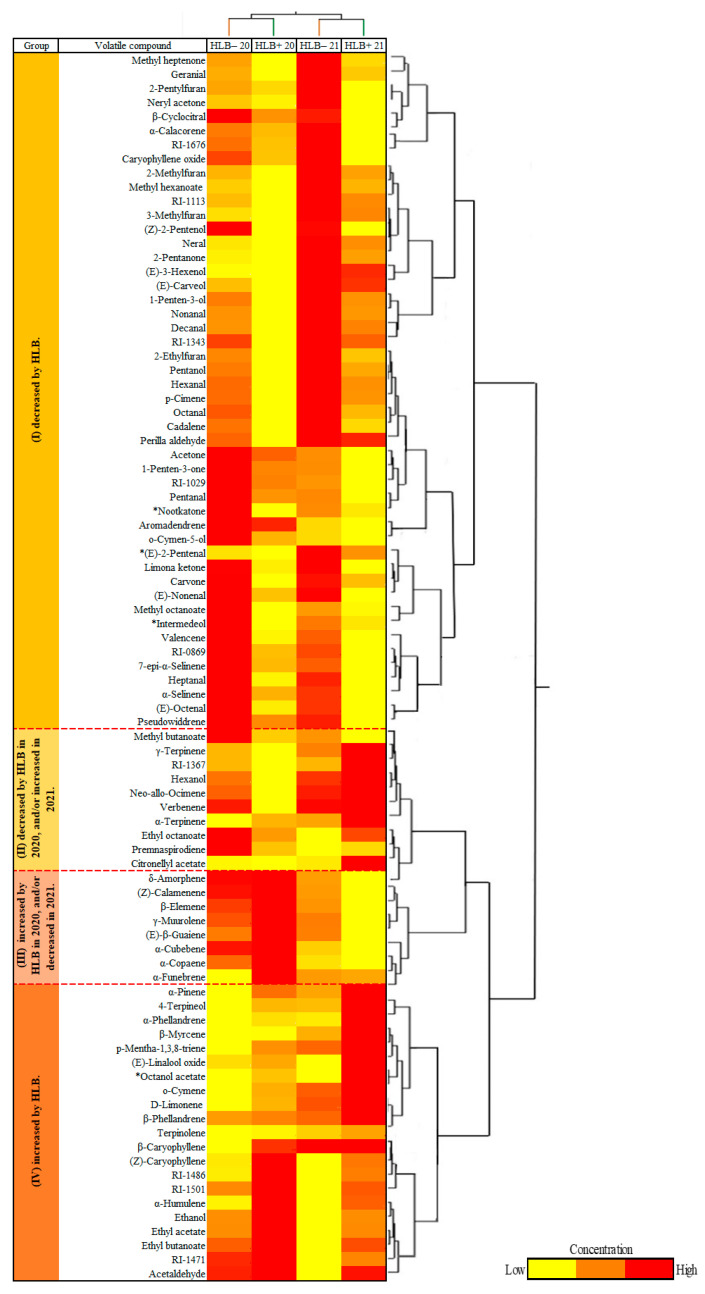
Combined cluster analysis and heat map of investigated grapefruit juice samples of healthy (HLB−) and HLB-affected fruits (HLB+) from 2020 (HLB− 20 and HLB+ 20) and 2021 (HLB− 21 and HLB+ 21) harvests (columns) and identified VOCs (rows). Chemical groups increased and/or decreased by Huanglongbing (HLB) determined the average concentration of volatiles in juice extracted from ‘Ray Ruby’ grapefruit in two harvest seasons, 2020 and 2021. * Significant differences at *p* ≤ 0.05 according to Tukey’s HSD test in both years.

The most abundant chemical class was monoterpene hydrocarbons with 13 components representing 47% to 55% of the total volatiles (Table 3). The major monoterpene hydrocarbons were D-limonene followed by β-myrcene (Table 3). Unlike in the peel oil, they both increased in the juice of HLB+ fruit, as well as many other monoterpene compounds (Figure 5), although the differences were not significant. β-myrcene has a strong green and metallic odor, which may negatively affect the aroma and flavor characteristics of citrus and orange juices when present in high amounts [11,40].

While sesquiterpene hydrocarbons constituted a small portion of the peel oil, 0.89–1.39% of total volatiles (Table 2), they were abundant in the juice, with relative abundances from 34 to 36% in 2020 and 31% in 2021 (Table 3). β-Caryophyllene has a woody–spicy, terpenic, sweet, and citrus flavor and is one of the key volatile components in grapefruit juice [41]. D-limonene and β-caryophyllene, the two major compounds in ‘Ray Ruby’ grapefruit juice volatiles, together accounted for nearly 70% of the total volatiles (Table 3). Both compounds decreased in HLB+ fruit in both harvest seasons (Figure 5). Limonene and β-caryophyllene are reported to decrease in juice as the fruit matures [42]. This could be the explanation for the higher concentration of these volatiles in HLB+ juice, considering the slow maturation of HLB+ fruits, which are usually similar to immature fruit [9].

These terpene hydrocarbons have chemo-preventative functions, such as induction of glutathione-S-transferase activity and uridine diphosphoglucuronosyl transferase activity in the small intestine and liver [42,43]. D-limonene also has chemotherapeutic activity against pancreatic, mammary, and prostatic tumors [44] and helps to prevent the initiation and suppress the progression of mammary and liver cancer [45].

**Figure 6:**
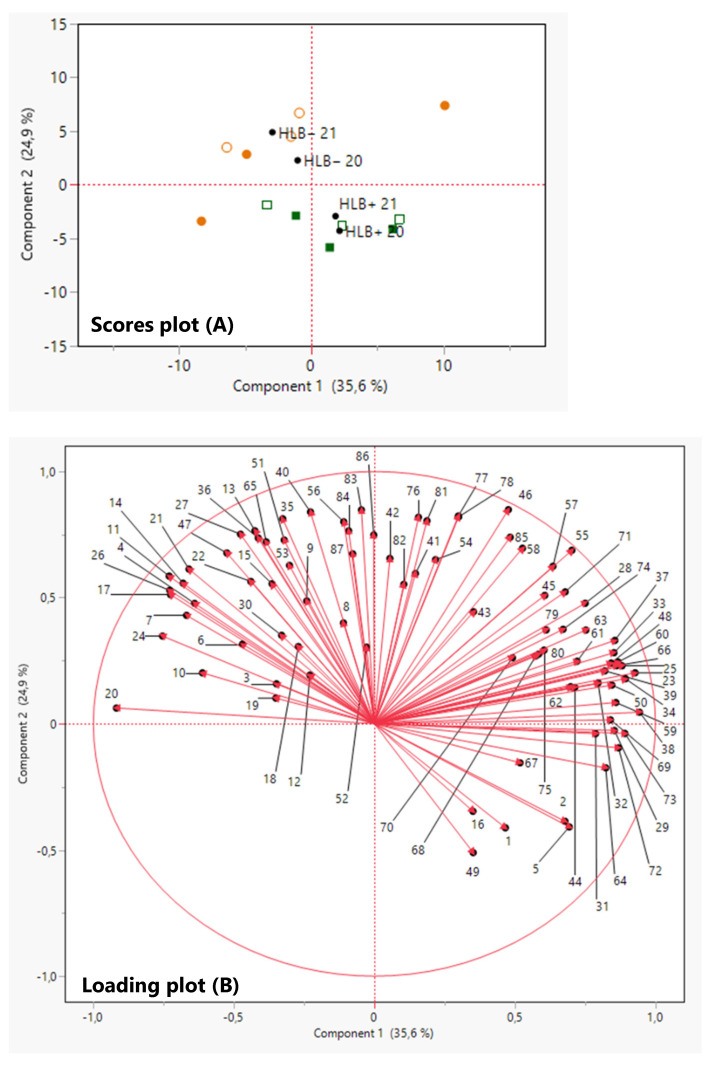
Principal component analysis (PCA) score plot (**A**) and loading plot (**B**) of VOCs in the juice of ‘Ray Ruby’ grapefruit of healthy (HLB−) and HLB-affected (HLB+) fruits from 2020 (HLB− 20 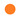 and HLB+ 20 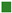) and 2021 (HLB− 21 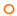 and HLB+ 21 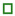) harvests. VOCs corresponding to the numbers are listed in Table 3.

Like in peel oil, nootkatone was significantly decreased in HLB+ fruit juice (Table 3, Figure 5 and Figure 6). Nootkatone concentration is considered the grapefruit juice quality indicator [33,34,39], and HLB negatively affected the juice flavor quality (Table 3, Figure 5 and Figure 6).

C8-C10 aldehydes were significantly lower in HLB+ fruit juice than in HLB− juice (Table 3, Figure 5 and Figure 6). These long-chain aldehydes and nootkatone are critical for high-quality grapefruit juice [36,42]. The results confirmed that HLB negatively affected the juice flavor quality (Table 3, Figure 5 and Figure 6).

The CA divided the compounds into four groups: group I with 48 out of 87 compounds decreased by HLB; group II with 10 compounds decreased by HLB in 2020 but increased by HLB in 2021; group III composed of 8 compounds increased by HLB in 2020 but decreased in 2021; and group IV with 21 compounds increased by HLB, including acetaldehydes, ethanol, and ethyl acetate (Figure 5). Contrary to peel oil, in which more VOCs were increased by HLB, more VOCs were decreased in HLB+ juice (48 decreases–21 increases, Figure 5).

HLB resulted in a significant reduction in aliphatic aldehydes and terpene ketones in the juice and an increase in aliphatic alcohols, aliphatic esters, and monoterpene hydrocarbons (Table 3, Figure 5). Long-chain aldehydes are known to contribute a lot to citrus juice aroma, with citrus-like, grassy, and soapy odors [33,46,47,48]. In grapefruit, ketones also strongly contribute to the flavor, mainly due to nootkatone, the main fragrant component of grapefruit [42,48]. On the other hand, ethanol is the aliphatic alcohol derived from the fermentation process, which is responsible for a fermented flavor in fresh fruit and juice [12,46]. The decrease of C8-C10 aldehydes and increased ethanol and esters could result in an “off-flavor” in the juice [46]. Valencene is considered the precursor of nootkatone, and the levels of both compounds have been reported to increase as the fruit matures [42,49]. The fact HLB decreased nootkatone in the peel oil and juice confirmed that HLB slowed the maturation of fruits, similar to immature orange fruits [9].

In Valencia oranges, valencene accumulates throughout fruit maturation, but in grapefruit, it is further oxidized to nootkatone [50]. On the other hand, we did not identify the presence of valencene in the peel oil (Table 2).

Consistent with our findings, previous studies also found that β-myrcene, D-limonene, and total terpenes had higher concentrations in HLB symptomatic Hamlin juice than healthy ones [8,9]. Previous studies also showed decreased levels of aldehyde compounds in Hamlin orange juice of HLB symptomatic fruits compared with the juice of healthy fruits [8,48].

PCA was performed to project the volatile juice components on a two-component plot (Figure 6). The PCA discriminated HLB+ from HLB− samples on Component 2, explaining 24.9% of the variation (Figure 6). Most juice volatiles were associated with HLB− samples, located on the positive side of PC2, while the VOCs compounds of HLB+ samples were on the negative side of PC2 (Figure 6).

**Figure 7:**
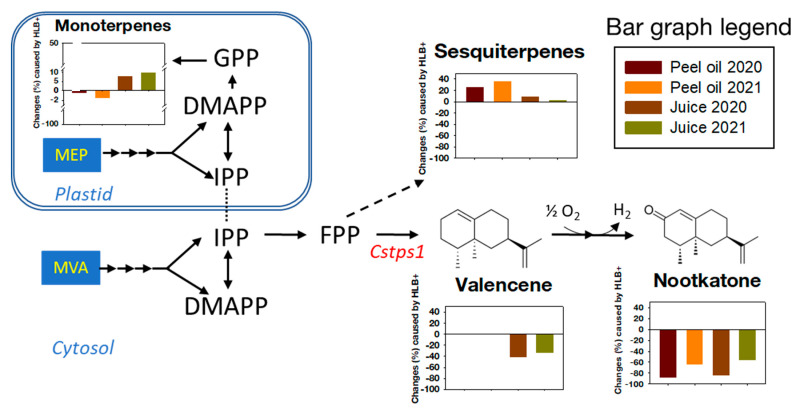
Summary of metabolic pathways leading to monoterpenes, sesquiterpenes, and nootkatones [49,52,53,55]. Bar graphs show changes (%) caused by HLB affection in peel oil and juice in 2020 and 2021. MEP, methylerythritol phosphate pathway; MVA, mevalonic acid pathway; IPP, isopentenyl pyrophosphate; DMAPP, dimethylallyl pyrophosphate; GPP, geranyl pyrophosphate; FPP, farnesyl pyrophosphate; Cstps1, valencene synthase.

HLB caused increases in sesquiterpenes and decreases in valencene and nootkatone in both peel oil and juice samples (Figure 7). Valencene is a major sesquiterpene in oranges and some mandarins, but it was not detectable in other mandarins [12,21,49,51]. Valencene existed in grapefruit juice samples, although it was not the major sesquiterpene (Table 3 and Figure 7). However, valencene was not detected in the peel oil samples, although there were other sesquiterpenes in the samples (Table 2 and Figure 7). Sharon-Asa et al. [52] reported that Cstps1 encodes valencene synthase, and Yu et al. [51] confirmed that the gene expression level of Cstps1 and the abundance of valencene were closely correlated. The opposite responses to HLB between total sesquiterpenes and valencene in juice samples confirm the above observation again, suggesting a possible mechanism that valencene biosynthesis may be independent of other sesquiterpenes (Figure 7).

The enzymatic steps from valencene to nootkatone in planta remain unclear [50,53]. However, in recent years, the integration of heterologous metabolic pathways indicates that regioselective oxidation of valencene at C-2 to β-nootkatol through cytochrome P450 was coupled with P450 reductase (CPR) and dehydrogenase-catalyzed oxidation of β-nootkatol to nootkatone [53,54]. Our data indicate that in peel tissue, nootkatone could be converted from nonvalencene sesquiterpenes, or that valencene is not accumulated in peel oil but is directly converted to nootkatone (Table 2 and Figure 7).

Monoterpenes are the major components in peel oil (about 97–98%) and juice (about 50%) samples (Table 2 and Table 3). HLB caused an increase of monoterpenes in peel oil samples but a decrease in juice samples (Figure 7, Table 2 and Table 3).

We focused on how HLB affects the volatile profiles in peel oil and juice. However, more complicated interactions mediated by volatiles exist among plants (including fruit), pathogens (including CLas), and insects (including the vector of CLas, ACP) [56,57,58,59]. Nootkatone, valencene, and many VOCs in citrus are insect repellents and/or antimicrobial agents [53,60,61]. The volatile profiling method has been used to compare the HLB tolerance of citrus cultivars [35,58,62]. Hijaz et al. [57] observed that the responses of the volatile profile in the citrus tree to ACP were more significant than to CLas infection. A better knowledge of the interactive effects of plant hosts and the insects and microbials on the volatile profile in the fruit may also help us better understand and estimate the tolerant plant selection, insect and disease control, and fruit quality control [56,59,63].

## 4. Conclusions

HLB decreased grapefruit quality, with smaller and greener fruit and a lower SSC and SSC/TA ratio. However, the responses of VOCs to HLB varied depending on the source (juice or peel oil) and harvest season. Nootkatone, the most important volatile component for grapefruit flavor, significantly decreased in juice and peel oil regardless of the harvest year. HLB disease resulted in a decrease of abundant monoterpenes, including D-limonene, β-myrcene, and α-pinene in the peel oil; opposite trends were found in the juice samples. On the other hand, the oxidative/dehydrogenated terpenes were increased by HLB in peel oil but decreased in the juice sample. The long chain aldehydes, decanal, nonanal, and octanal also increased in HLB peel oil but decreased in the juice, resulting in likely lower flavor quality in the juice. Our results indicated that the volatile profiles of peel oil and juice had different responses to HLB than those in sweet orange fruit. This may affect the decision of fruit processors to favor processing for peel oil or juice depending on the fruit source received at the processing plant.

## Figures and Tables

**Table 1:** Quality attributes of ‘Ray Ruby’ grapefruit and juice of healthy (HLB−) and HLB-affected (HLB+) fruits of two harvest seasons, 2020 and 2021.

Parameter	2020			2021		
	HLB−	HLB+	*p*-Value	HLB−	HLB+	*p*-Value
Fruit weight (g)	482.8 a	230.8 b	<0.001 ***	500.6 a	228.8 b	<0.001 ***
Fruit length (mm)	89.3 a	74.0 b	<0.001 ***	96.7a	74.8 b	<0.001 ***
Fruit diameter (mm)	104.8 a	79.9 b	<0.001 ***	99.9 a	81.2 b	<0.001 ***
Peel a*	8.50 a	−7.25 b	<0.001 ***	8.56	−0.19	0.058
Peel b*	42.62	46.54	0.053	45.13	52.89	0.083
Peel a*/b* ratio	0.199 a	−0.156 b	<0.001 ***	0.190 a	−0.036 b	<0.001 ***
Hue angle (°)	78.8 b	98.8 a	<0.001 ***	79.3 b	92.3 a	<0.001 ***
SSC (%)	10.77 a	9.50 b	0.009 ***	9.63 a	8.35 b	0.008 ***
TA (%)	1.10 a	1.15 a	0.408	0.89 a	0.99 a	0.347
SSC/TA ratio	9.79 a	8.28 b	0.029 *	10.93 a	8.61 b	0.042 *

* and *** indicate a significant difference between HLB− and HLB+ according to the Tukey test at *p* ≤ 0.05 and 0.001, respectively. Different lowercase letters indicate a significant difference between HLB− and HLB+ (*p* ≤ 0.05).

**Table 2:** Chemical composition (%) of the peel oil of ‘Ray Ruby’ grapefruit of healthy (HLB−) and HLB-affected (HLB+) fruits from 2020 and 2021 harvests.

Peak No.	VOC	Calculated RI ^z^	Reference RI ^y^	2020				2021			
				HLB−	HLB+	*p*-Value	Difference ^x^	HLB−	HLB+	*p*-Value	Difference
1	Hexanal	795	805	0.001	0.003	0.0124 *	200.00%	0.004	0.003	0.2907	−25.00%
2	(*E*)-2-Hexenal	852	853	0.003	0.008	0.0058 *	166.70%	0.014	0.009	0.3918	−35.70%
3	(*E*)-3-Hexenol	855	855	0.000 ^w^	0	- ^v^	-	0.017	0.013	0.4063	−23.50%
4	Heptanal	902	903	0	0	-	-	0	0.001	0.0091 *	++ ^u^
5	α-Thujene	937	932	0.002	0.003	0.3252	50.00%	0.003	0.003	0.546	0.00%
6	α-Pinene	949	940	0.724	0.587	<0.001 *	−18.90%	0.689	0.638	0.0120 *	−7.40%
7	Camphene	967	951	0.003	0.003	0.0585	0.00%	0.004	0.003	0.1556	−25.00%
8	Sabinene	988	976	0.2	0.256	0.0195 *	28.00%	0.336	0.352	0.6949	4.80%
9	β-Myrcene	997	990	2051	1744	0.0004 *	−15.00%	1,949	1836	0.0025 *	−5.80%
10	Octanal	1007	1003	0.09	0.218	0.0119 *	142.20%	0.244	0.257	0.7337	5.30%
11	Hexyl acetate	1013	1006	0.002	0	0.0005 *	−100.00%	0.002	0.002	0.3624	0.00%
12	α-Phellandrene	1019	1010	0.045	0.036	0.0024 *	−20.00%	0.045	0.042	0.0516	−6.70%
13	α-Terpinene	1031	1020	0.004	0.005	0.1954	25.00%	0.008	0.011	0.1022	37.50%
14	D-Limonene	1053	1030	93,782	93,709	0.7855	−0.10%	94,799	93,529	0.4629	−1.30%
15	β-Phellandrene	1054	1031	0.181	0.178	0.5735	−1.70%	0.18	0.179	0.9456	−0.60%
16	β-Ocimene	1054	1035	0.081	0.115	0.0072 *	42.00%	0.079	0.137	0.0036 *	73.40%
17	γ-Terpinene	1072	1062	0.01	0.014	0.0779	40.00%	0.083	0.054	0.5522	−34.90%
18	(*Z*)-Linalool oxide	1086	1080	0.304	0.265	0.3216	−12.80%	0.195	0.242	0.1633	24.10%
19	(*E*)-Linalool oxide	1101	1086	0.152	0.138	0.4642	−9.20%	0.101	0.123	0.2279	21.80%
20	Terpinolene	1103	1088	0.013	0.019	0.1568	46.20%	0.015	0.02	0.1705	33.30%
21	Linalool	1106	1101	0.136	0.165	0.102	21.30%	0.138	0.176	0.297	27.50%
22	Nonanal	1108	1102	0.035	0.047	0.0802	34.30%	0.059	0.054	0.4627	−8.50%
23	(*E*)-4,8-Dimethylnona-1,3,7-triene	1120	1116	0.012	0.011	0.2153	−8.30%	0.009	0.01	0.7871	11.10%
24	Methyl octanoate	1124	1126	0.004	0.007	0.0122 *	75.00%	0	0	-	-
25	p-Mentha-1,3,8-triene	1127	1118	0	0.001	0.0054 *	++	0	0.002	0.0001 *	++
26	(*E*)-p-Mentha-2,8-dien-1-ol	1137	1128	0.021	0.024	0.5517	14.30%	0.013	0.02	0.25	53.80%
27	(*Z*)-p-Mentha-2,8-dien-1-ol	1151	1138	0.013	0.015	0.2621	15.40%	0.011	0.016	0.2967	45.50%
28	(*E*)-Limonene oxide	1153	1139	0.013	0.014	0.6275	7.70%	0.008	0.01	0.5024	25.00%
29	Citronellal	1158	1152	0.038	0.068	0.0268 *	78.90%	0.042	0.076	0.0309 *	81.00%
30	Octanoic acid	1160	1180	0.017	0.051	0.0050 *	200.00%	0	0	-	-
31	Ethyl octanoate	1194	1195	0.011	0.01	0.8238	−9.10%	0	0	-	-
32	4-Terpinenol	1197	1182	0.025	0.03	0.2734	20.00%	0.029	0.043	0.1799	48.30%
33	(*E*)-p-mentha-1(7),8-dien-2-ol	1206	1204	0	0	-	-	0.005	0.009	0.2007	80.00%
34	Decanal	1208	1205	0.351	0.391	0.2134	11.40%	0.414	0.379	0.4399	−8.50%
35	(*Z*) Dihydro carvone	1214	1210	0.008	0.008	0.7397	0.00%	0.005	0.009	0.1795	80.00%
36	(*Z*)-Isopiperitenol	1220	1228	0	0	-	-	0.01	0.018	0.1877	80.00%
37	(*E*) Dihydro carvone	1223	1230	0.008	0.007	0.5321	−12.50%	0.004	0.005	0.1548	25.00%
38	Neral	1248	1240	0.031	0.061	0.067	96.80%	0.038	0.039	0.937	2.60%
39	Geraniol	1261	1250	0	0	-	-	0.011	0.005	0.0033 *	−54.50%
40	Carvone	1263	1242	0.021	0.029	0.0214 *	38.10%	0.017	0.016	0.7048	−5.90%
41	1-Decanol	1275	1270	0	0	-	-	0.006	0.006	0.665	0.00%
42	Geranial	1277	1271	0.03	0.055	0.0178 *	83.30%	0.062	0.062	0.9822	0.00%
43	Perilla aldehyde	1295	1272	0.016	0.031	0.0004 *	93.80%	0.015	0.031	0.2256	106.70%
44	Undecanal	1307	1305	0.018	0.01	0.1639	−44.40%	0.014	0.012	0.2492	−14.30%
45	Decanoic acid	1351	1370	0.007	0.011	0.0411 *	57.10%	0	0.002	0.0235 *	++
46	γ-Terpineol	1359	1200	0.019	0.018	0.7496	−5.30%	0.017	0.021	0.3144	23.50%
47	(*Z*)-Carvyl acetate	1362	1360	0.017	0.018	0.4091	5.90%	0.016	0.024	0.1349	50.00%
48	α-Cubebene	1369	1361	0.007	0.009	0.0546	28.60%	0.006	0.009	0.0313 *	50.00%
49	Geranyl acetate	1377	1380	0.083	0.062	0.0141 *	−25.30%	0.084	0.074	0.4158	−11.90%
50	Ethyl decanoate	1387	1391	0.002	0.001	0.0190 *	−50.00%	0	0	-	-
51	α-Copaene	1402	1380	0.151	0.197	0.0175 *	30.50%	0.134	0.162	0.0181 *	20.90%
52	Dodecanal	1407	1405	0.02	0.024	0.0671	20.00%	0.025	0.024	0.6882	−4.00%
53	β-Cubebene	1413	1426	0.136	0.172	0.0149 *	26.50%	0.124	0.149	0.0124 *	20.20%
54	p-Mentha-1,8-dien-7-yl acetate	1442	1436	0.004	0.004	0.757	0.00%	0.005	0.005	0.9257	0.00%
55	β-Caryophyllene	1454	1420	0.412	0.501	0.0297 *	21.60%	0.304	0.464	0.0098 *	52.60%
56	(*Z*)-β-Farnesene	1457	1443	0.012	0.017	0.2619	41.70%	0.019	0.025	0.4055	31.60%
57	β-Copaene	1460	1430	0.005	0.006	0.7414	20.00%	0.005	0.007	0.0813	40.00%
58	α-Guaiene	1462	1440	0.005	0.006	0.7193	20.00%	0.005	0.007	0.0080 *	40.00%
59	α-Humulene	1489	1455	0.064	0.076	0.1021	18.80%	0.047	0.066	0.0221 *	40.40%
60	γ-Muurolene	1502	1477	0	0.01	<0.0001 *	++	0.006	0.009	0.0066 *	50.00%
61	(*E*)-α-Farnesene	1508	1506	0.011	0.023	0.0657	109.10%	0	0	-	-
62	Germacrene D	1515	1482/1519	0.08	0.103	0.08	28.80%	0.063	0.079	0.0299 *	25.40%
63	α-Muurolene	1525	1500	0.027	0.028	0.741	3.70%	0.013	0.02	0.0011 *	53.80%
64	Bicyclogermacrene	1531	1502	0.024	0.026	0.7317	8.30%	0.014	0.02	0.0298 *	42.90%
65	α-Bulnesene	1535	1510	0.003	0.004	0.06	33.30%	0.003	0.009	0.3503	200.00%
66	β-Sesquiphellandrene	1540	1521	0	0	-	-	0	0.003	0.0096 *	++
67	δ-Cadinene	1547	1523	0.177	0.211	0.0179 *	19.20%	0.148	0.184	0.0455 *	24.30%
68	(*E)*-Nerolidol	1570	1535	0.028	0.017	0.0191 *	−39.30%	0.009	0.01	0.3918	11.10%
69	Elemol	1577	1551	0.041	0.045	0.5934	9.80%	0.022	0.025	0.6186	13.60%
70	Caryophyllene oxide	1630	1580	0.024	0.017	0.1633	−29.20%	0.012	0.011	0.1373	−8.30%
71	γ-Eudesmol	1675	1622	0	0.008	0.0138 *	++	0.005	0.005	0.5735	0.00%
72	α-Murrolol	1686	1642	0	0	-	-	0.012	0.011	0.981	−8.30%
73	α-Cadinol	1695	1653	0.009	0.012	0.1668	33.30%	0.007	0.007	0.8972	0.00%
74	β -Sinensal	1719	1706	0.02	0.026	0.2684	30.00%	0.017	0.023	0.4113	35.30%
75	Nootkatone	1843	1820	0.127	0.016	0.0264 *	−87.40%	0.019	0.007	0.0059 *	−63.20%
	**Chemical class**										
	Monoterpene hydrocarbons			97.11	96.68	0.1437	−0.40%	98.2	96.81	0.4154	−1.40%
	Sesquiterpene hydrocarbons			1.11	1.39	0.0194 *	25.20%	0.89	1.21	0.0155 *	36.00%
	Aliphatic aldehydes			0.52	0.7	0.0149*	34.60%	0.78	0.74	0.9413	−5.10%
	Terpene aldehydes			0.13	0.24	0.0816	84.60%	0.17	0.23	0.2666	35.30%
	Total aldehydes			0.65	0.94	0.0096 *	44.60%	0.95	0.97	0.8814	2.10%
	Terpene oxides			0.49	0.44	0.3522	−10.20%	0.32	0.39	0.1933	21.90%
	Alcohols			0.29	0.33	0.1597	13.80%	0.31	0.38	0.3717	22.60%
	Ketones			0.16	0.06	0.0437 *	−62.50%	0.04	0.04	0.3921	0.00%
	Esters			0.12	0.1	0.0377 *	−16.70%	0.11	0.1	0.8718	−9.10%

^z^ Calculated RI: retention index calculated from the retention time of each volatile and n-alkanes on the DB-5 column. ^y^ Reference RI: RI based on NIST GC RI, 5% equivalence nonpolar column (such as DB-5, BP-5, and HP-5). ^x^ HLB+ caused positive or negative changes. ^w^ composition less than 0.0005%. ^v^ “-” not available. ^u^ “++” compound induced by HLB+. * Significant difference at *p* ≤ 0.05 according to Tukey’s HSD test.

**Table 3:** Signal intensity (peak size, total ion current ×10^7^) of volatile organic compounds of ‘Ray Ruby’ grapefruit juice of healthy (HLB−) and HLB-affected (HLB+) fruits from 2020 and 2021 harvests.

Peak No.	VOC	Calculated RI ^z^	Reference RI ^y^	2020				2021			
				HLB–	HLB+	*p*-Value	Difference ^x^	HLB–	HLB+	*p*-Value	Difference
1	Acetaldehyde	447	380	1.37	1.49	0.6811	8.8%	0.61	1.41	0.2879	131.1%
2	Ethanol	475	450	5.05	9.94	0.1798	96.8%	1.03	5.06	0.0570	391.3%
3	Acetone	500	500	1.67	1.48	0.745	−11.4%	1.38	1.15	0.2660	−16.7%
4	2-Methylfuran	595	605	0.66	0.54	0.1301	−18.2%	0.96	0.70	0.2193	−27.1%
5	Ethyl acetate	600	610	3.05	5.62	0.1847	84.3%	0.94	3.11	0.0819	230.9%
6	3-Methylfuran	606	620	0.91	0.88	0.5746	−3.3%	1.08	0.98	0.6796	−9.3%
7	1-Penten-3-ol	674	680	0.15	0.09	0.1767	−40.0%	0.21	0.14	0.0505	−33.3%
8	1-Penten-3-one	678	685	0.61	0.43	0.0676	−29.5%	0.42	0.26	0.0378 *	−38.1%
9	2-Pentanone	689	695	0.17	0.13	0.3553	−23.5%	0.75	0.37	0.1375	−50.7%
10	Pentanal	692	700	3.71	1.97	0.1968	−46.9%	2.11	0.66	0.0084 *	−68.7%
11	2-Ethylfuran	696	705	0.40	0.21	0.1229	−47.5%	0.60	0.30	0.1005	−50.0%
12	Methyl butanoate	715	720	0.28	0.18	0.2831	−35.7%	0.20	0.15	0.1980	−25.0%
13	(*E*)-2-Pentenal	754	750	0.16	0.00 ^v^	<0.0001 *	−100.0%	0.15	0.00	<0.0001 *	−100.0%
14	Pentanol	761	765	0.24	0.11	0.053	−54.2%	0.36	0.20	0.0640	−44.4%
15	(*Z*)-2-Pentenol	765	770	0.15	0.09	0.0172 *	−40.0%	0.61	0.32	0.0981	−47.5%
16	Ethyl butanoate	796	800	1.80	2.39	0.5098	32.8%	0.79	1.90	0.1482	140.5%
17	Hexanal	801	805	41.45	15.93	0.0479 *	−61.6%	59.57	35.27	0.1921	−40.8%
18	(*E*)-3-Hexenol	857	855	1.77	1.69	0.7959	−4.5%	5.88	5.14	0.6759	−12.6%
19	Hexanol	867	865	0.94	0.65	0.2656	−30.9%	1.07	1.18	0.6968	10.3%
20	RI-0869 ^w^	869	-	3.22	2.76	0.4301	−14.3%	3.04	2.60	0.2134	−14.5%
21	Heptanal	905	903	6.89	2.33	0.0198 *	−66.2%	6.18	2.11	0.0050 *	−65.9%
22	Methyl hexanoate	922	925	1.15	0.69	0.1551	−40.0%	2.98	1.38	0.1128	−53.7%
23	α-Pinene	954	940	2.97	3.53	0.7586	18.9%	3.32	3.96	0.6181	19.3%
24	Methyl heptenone	986	986	56.75	51.22	0.3349	−9.7%	66.09	53.49	0.3871	−19.1%
25	β-Myrcene	993	990	33.95	34.12	0.9944	0.5%	39.68	51.72	0.4990	30.3%
26	2-Pentylfuran	996	992	0.53	0.43	0.6037	−18.9%	0.85	0.35	0.0245 *	−58.8%
27	Octanal	1006	1003	2.62	0.98	0.0016 *	−62.6%	3.45	1.67	0.0629	−51.6%
28	Verbenene	1021	956	0.68	0.53	0.6212	−22.1%	0.69	0.70	0.9706	1.4%
29	α-Phellandrene	1023	1010	0.55	0.61	0.8729	10.9%	0.59	1.07	0.2119	81.4%
30	RI-1029	1029	-	0.24	0.17	0.2728	−29.2%	0.16	0.09	0.2245	−43.8%
31	α-Terpinene	1033	1020	0.67	0.86	0.6049	28.4%	0.90	1.30	0.3318	44.4%
32	o-Cymene	1040	1023	3.39	3.84	0.6628	13.3%	4.28	4.79	0.4519	11.9%
33	D-Limonene	1048	1030	404.14	437.84	0.8006	8.3%	480.82	517.63	0.6770	7.7%
34	β-Phellandrene	1052	1031	3.22	3.59	0.8804	11.5%	3.96	5.35	0.4181	35.1%
35	p-Cimene	1057	1036	0.83	0.53	0.0172 *	−36.1%	1.04	0.75	0.0360 *	−27.9%
36	(*E*)-Octenal	1062	1060	0.83	0.30	0.0023 *	−63.9%	0.71	0.26	0.0122 *	−63.4%
37	γ-Terpinene	1071	1062	0.58	0.52	0.8246	−10.3%	0.62	0.73	0.5986	17.7%
38	(*E*)-Linalool oxide	1084	1086	0.86	0.94	0.849	9.3%	0.81	1.19	0.1313	46.9%
39	Terpinolene	1101	1088	1.87	2.02	0.8953	8.0%	2.53	3.14	0.4702	24.1%
40	Nonanal	1105	1102	4.64	3.64	0.1575	−21.6%	5.93	4.59	0.0027 *	−22.6%
41	RI-1113	1113	-	0.31	0.25	0.4404	−19.4%	0.48	0.36	0.2803	−25.0%
42	Methyl octanoate	1117	1126	0.71	0.33	0.0459 *	−53.5%	0.49	0.35	0.2712	−28.6%
43	o-Cymen-5-ol	1125	-	0.71	0.50	0.4011	−29.6%	0.46	0.42	0.6126	−8.7%
44	p-Mentha-1,3,8-triene	1128	1118	0.15	0.17	0.5552	13.3%	0.18	0.20	0.5945	11.1%
45	Neo-allo-Ocimene	1145	1131	0.08	0.05	0.3697	−37.5%	0.09	0.09	0.7875	0.0%
46	Limona ketone	1147	1144	0.27	0.19	0.3673	−29.6%	0.27	0.18	0.0527	−33.3%
47	(*E*)-Nonenal	1162	1162	0.32	0.16	0.1569	−50.0%	0.32	0.11	0.0027 *	−65.6%
48	Ethyl octanoate	1187	1195	2.29	1.47	0.658	−35.8%	0.92	1.90	0.3276	106.5%
49	Octanol acetate	1201	1211	0.00	0.10	0.0283 *	++ ^u^	0.00	0.41	0.0195 *	++
50	4-Terpineol	1203	1182	0.21	0.25	0.8277	19.0%	0.25	0.35	0.4555	40.0%
51	Decanal	1206	1205	0.43	0.24	0.1113	−44.2%	0.69	0.46	0.1254	−33.3%
52	(*E*)-Carveol	1235	1217	0.33	0.21	0.4553	−36.4%	0.68	0.58	0.8259	−14.7%
53	β-Cyclocitral	1244	1223	1.05	0.84	0.4535	−20.0%	1.00	0.68	0.0719	−32.0%
54	Neral	1247	1240	0.40	0.37	0.7952	−7.5%	0.64	0.49	0.0114 *	−23.4%
55	Carvone	1266	1242	1.20	0.77	0.5385	−35.8%	1.16	0.88	0.2813	−24.1%
56	Geranial	1274	1271	0.85	0.69	0.2611	−18.8%	1.18	0.80	0.0153 *	−32.2%
57	Perilla aldehyde	1306	1272	0.10	0.06	0.5129	−40.0%	0.12	0.11	0.8090	−8.3%
58	RI-1343	1343	-	0.39	0.17	0.3146	−56.4%	0.46	0.35	0.4882	−23.9%
59	Citronellyl acetate	1348	1355	0.29	0.28	0.9678	−3.4%	0.30	0.55	0.2422	83.3%
60	RI-1367	1367	-	0.33	0.26	0.797	−21.2%	0.33	0.50	0.4228	51.5%
61	α-Cubebene	1381	1361	3.28	3.47	0.9063	5.8%	1.96	1.59	0.5229	−18.9%
62	α-Copaene	1423	1380	11.31	13.93	0.6077	23.2%	8.28	7.48	0.7582	−9.7%
63	β-Elemene	1429	1384	3.34	3.55	0.8735	6.3%	3.06	2.70	0.5631	−11.8%
64	(*Z*)-Caryophyllene	1463	1405	2.21	2.96	0.2626	33.9%	2.14	2.58	0.1100	20.6%
65	Neryl acetone	1466	1434	1.61	1.35	0.1231	−16.1%	2.79	1.27	0.0002 *	−54.5%
66	RI-1471	1471	-	0.79	0.81	0.9556	2.5%	0.69	0.75	0.4549	8.7%
67	α-Funebrene	1476	1385	0.25	0.40	0.0358 *	60.0%	0.31	0.30	0.8610	−3.2%
68	β-Caryophyllene	1482	1420	234.29	248.18	0.606	5.9%	245.09	251.65	0.7335	2.7%
69	RI-1486	1486	-	9.00	10.43	0.6083	15.9%	7.74	9.49	0.1092	22.6%
70	Aromadendrene	1497	1440	0.57	0.51	0.7684	−10.5%	0.26	0.21	0.4499	−19.2%
71	Premnaspirodiene	1498	1505	0.47	0.42	0.6351	−10.6%	0.40	0.41	0.7714	2.5%
72	RI-1501	1501	-	2.82	3.86	0.3824	36.9%	2.74	3.31	0.1840	20.8%
73	α-Humulene	1514	1455	37.54	51.31	0.461	36.7%	36.83	45.93	0.2014	24.7%
74	γ-Muurolene	1520	1477	1.57	1.83	0.7173	16.6%	1.44	1.02	0.2465	−29.2%
75	(*E*)-β-Guaiene	1532	1490	0.52	0.64	0.6221	23.1%	0.51	0.38	0.4463	−25.5%
76	Pseudowiddrene	1538	1523	4.86	3.74	0.4153	−23.0%	4.59	2.78	0.0423 *	−39.4%
77	Valencene	1546	1492	4.58	2.69	0.2728	−41.3%	3.86	2.61	0.1532	−32.4%
78	α-Selinene	1551	1496	2.51	1.81	0.385	−27.9%	2.28	1.49	0.0954	−34.6%
79	δ-Amorphene	1558	1512	14.79	15.08	0.9574	2.0%	11.04	8.51	0.4020	−22.9%
80	(*Z*)-Calamenene	1566	1518	1.85	1.91	0.8863	3.2%	1.46	1.17	0.2506	−19.9%
81	7-epi-α-Selinene	1578	1520	3.15	1.98	0.2758	−37.1%	2.56	1.53	0.0370 *	−40.2%
82	α-Calacorene	1589	1542	0.31	0.26	0.7017	−16.1%	0.40	0.21	0.2672	−47.5%
83	Caryophyllene oxide	1598	1580	2.56	2.03	0.3732	−20.7%	2.85	1.77	0.0124 *	−37.9%
84	RI-1676	1676	-	1.16	0.98	0.5716	−15.5%	1.38	0.85	0.0106 *	−38.4%
85	Cadalene	1694	1674	0.39	0.33	0.5114	−15.4%	0.44	0.35	0.2595	−20.5%
86	Intermedeol	1701	1675	1.95	0.50	0.0435 *	−74.4%	1.27	0.65	0.0118 *	−48.8%
87	Nootkatone	1849	1820	4.30	0.71	0.0467 *	−83.5%	2.35	1.05	0.0166 *	−55.3%
	**Chemical class**										
	Monoterpene hydrocarbons			453.25	488.29	0.8315	7.7%	538.85	591.71	0.6357	9.8%
	Sesquiterpene hydrocarbons			327.8	354.98	0.6445	8.3%	326.91	332.9	0.8463	1.8%
	Terpene alcohols			3.2	1.46	0.1034	−54.4%	2.66	2	0.1868	−24.8%
	Terpene aldehydes			2.39	1.96	0.3414	−18.0%	2.94	2.08	0.0023 *	−29.3%
	Terpene ketones			5.77	1.67	0.0495 *	−71.1%	3.79	2.11	0.0345 *	−44.3%
	Terpene esters			2.29	1.57	0.697	−31.4%	0.92	2.32	0.1967	152.2%
	Terpene oxide			3.56	3.14	0.6047	−11.8%	3.83	3.17	0.2033	−17.2%
	Total terpene compounds			798.25	853.06	0.8096	6.9%	880.59	936.75	0.6463	6.4%
	Aliphatic aldehydes			62.42	27.04	0.0334 *	−56.7%	79.72	46.54	0.0489 *	−41.6%
	Aliphatic alcohols			8.31	12.57	0.2115	51.3%	9.16	12.04	0.175	31.4%
	Aliphatic ketones			60.81	54.61	0.3009	−10.2%	71.42	56.54	0.3248	−20.8%
	Aliphatic esters			7.28	9.48	0.4531	30.2%	5.7	7.44	0.278	30.5%
	Furan			2.5	2.06	0.2469	−17.6%	3.5	2.33	0.1537	−33.4%
	Total non-terpene compounds			141.32	105.76	0.0498 *	−25.2%	168.8	124.42	0.2137	−26.3%
	Other			17.93	19.42	0.6998	8.3%	16.7	17.8	0.4713	6.6%
	Total			957.5	978.25	0.9243	2.2%	1066.09	1078.97	0.8944	1.2%

^z^ Calculated RI: retention index calculated from the retention time of each volatile and n-alkanes on the DB-5 column. ^y^ Reference RI: RI based on NIST GC RI, 5% equivalence nonpolar column (such as DB-5, BP-5, and HP-5). ^x^ HLB+ caused positive or negative changes. ^w^ Unknown compound labeled with “RI” plus a four-digit retention index of the peak. ^v^ peak size less than 50,000 total ion currents. ^u^ ++: compound induced by HLB+. * Significant difference at *p* ≤ 0.05 according to Tukey’s HSD test.

## Data Availability

Data are contained within the article.

## Supplementary Materials

The following supporting information can be downloaded at: https://www.mdpi.com/article/10.3390/foods12040713/s1, Figure S1: GC-MS chromatograms of peel oil samples extracted from HLB+ (top) and HLB− (bottom) ‘Ray Ruby’ grapefruit; Figure S2: GC-MS chromatograms of juice extracted from HLB+ (top) and HLB− (bottom) ‘Ray Ruby’ grapefruit.
