# Supplementary material for: Effect of Huanglongbing on the Volatile Organic Compound Profile of Fruit Juice and Peel Oil in ‘Ray Ruby’ Grapefruit
Source: Foods. 2023 Feb 7;12(4):713. doi: 10.3390/foods12040713 (PMC9955810; doi:10.3390/foods12040713)
Supplement: Supplementary file 1 [file foods-12-00713-s001.zip › foods-2071122-supplementary.pdf]

foods MDPI

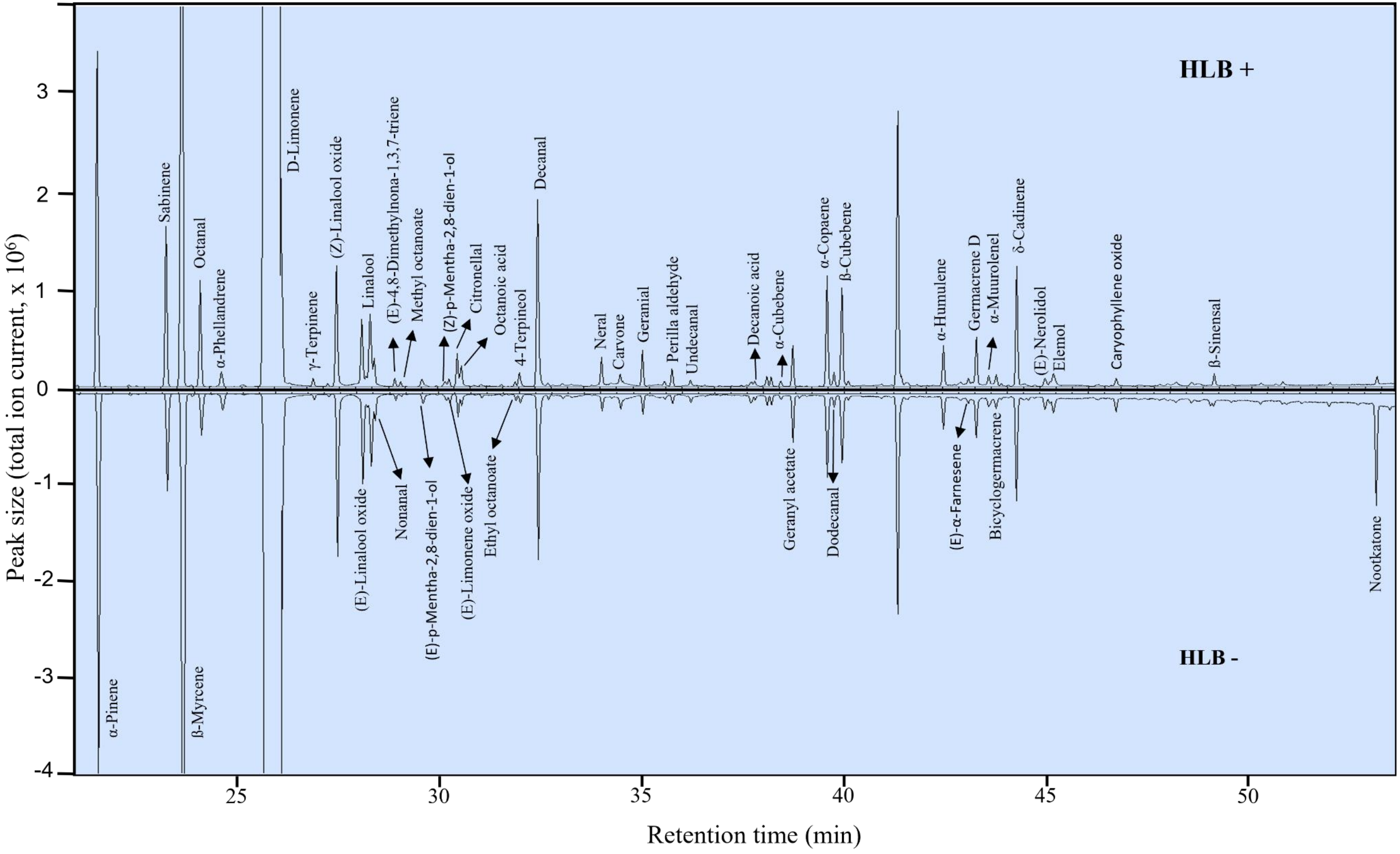

Figure S1. GC-MS chromatograms of peel oil samples extracted from HLB+ (top) and HLB- (bottom) 'Ray Ruby' grapefruit.

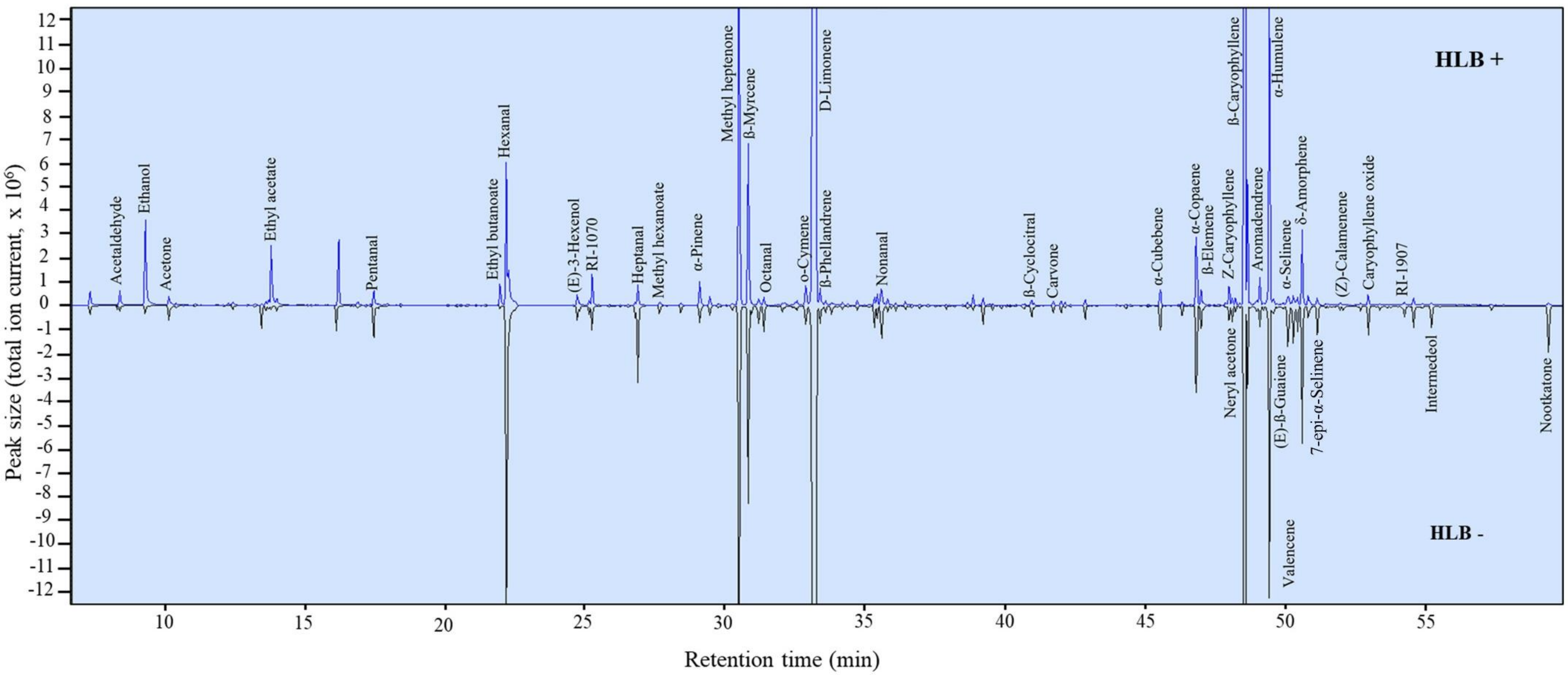

Figure S2. GC-MS chromatograms of juice extracted from HLB+ (top) and HLB- (bottom) 'Ray Ruby' grapefruit.
